# Behavioral convergence under urbanization: An overlooked dimension of biotic homogenization

**DOI:** 10.1371/journal.pbio.3003689

**Published:** 2026-03-02

**Authors:** Peter Mikula, Daniel T. Blumstein, Piotr Tryjanowski

**Affiliations:** 1 Faculty of Environmental Sciences, Czech University of Life Sciences Prague, Prague, Czech Republic; 2 Department of Ecology and Evolutionary Biology, University of California, Los Angeles, California, United States of America; 3 Department of Zoology, Poznań University of Life Sciences, Poznań, Poland

## Abstract

A variety of human activities, especially urbanization, are not only homogenizing species composition but also eroding behavioral diversity. This Essay introduces the concept of behavioral homogenization: the human-driven convergence of behavioral traits across individuals, populations, and species across space and time. Global examples of fear responses, foraging, communication, activity patterns, social behavior, cognition and exploration, habitat use, breeding-site choice, migration, and heterospecific interaction networks are used to argue that spatial and temporal beta-diversity in behavior is shrinking in human-dominated landscapes. Ecological and evolutionary consequences, including for animal cultures and human–wildlife conflict, are outlined and opportunities to quantify and integrate behavioral homogenization into biodiversity conservation and management are highlighted.

## Abstract 2

A consequence of Anthropogenic change is behavioral homogenization: the human-driven convergence of behavioral traits across individuals, populations, and species across space and time, often in urban environments. This Essay describes these changes and discusses their ecological and evolutionary consequences.

## Introduction

Human-driven biotic homogenization—the convergence of once-distinct biotas, for example, as driven by urbanization—erodes spatiotemporal community uniqueness [1,2]. Yet, the prevailing focus on species richness and composition largely overlooks a parallel axis of change: behavior. Behavior is the phenotype by which organisms interact with their environment, mediating species interactions, community dynamics, ecosystem function, and evolutionary trajectories [3]. Hence, integrating behavior into studies of global homogenization will be essential to produce a more comprehensive understanding of the topic, including its conservation and management consequences. For example, anthropogenic behavioral homogenization can erode the behavioral diversity that buffers animal populations and communities against further change [4], reshape interaction networks and ecosystem functioning [5], and increase human–wildlife conflict [6]. Despite its importance, behavioral homogenization remains relatively underexplored owing to the focus on behavioral plasticity and the often high within- and among-population variance. In addition, behavioral data has, until recently, been relatively more difficult to obtain than species counts, morphology, development, and other types of data traditionally used in studies of biotic homogenization [1,2].

In this Essay, we define behavioral homogenization and its key components, synthesize evidence across major behavioral axes, and outline ecological, evolutionary, conservation, and management implications under urbanization. We use the term urbanization broadly to mean the expansion of urban land cover and infrastructure; for simplicity and comparability, we focus primarily on clear urban–rural comparisons, while also drawing on studies that leverage gradients in human disturbance and time since urban colonization to capture the spatial and temporal components of behavioral homogenization. Crucially, homogenizing pressures are not confined to city centers; the spread of human activities and infrastructure drives behavior homogenization at much broader spatial scales. The processes we describe most probably do not stop at city boundaries and may propagate into rural or semi-natural environments.

## Behavioral homogenization

Behavioral homogenization is the process whereby behaviors become more uniform (less variable/diverse), often in response to shared human-driven selection pressures. In other words, populations or species that once exhibited distinct behavioral responses to the same stimuli in different regions begin to converge on behavioral patterns. We argue that behavioral homogenization emerges via two distinct processes: a spatial aspect (i.e., declining behavioral diversity among sites) and a temporal aspect (i.e., progressive within-population canalization and loss of behavioral variance with continued urban exposure). Moreover, behavioral homogenization is working at two taxonomic scales: within-species (i.e., same-species populations in different cities become more similar) and among-species (i.e., variously related species become convergent in behavior).

Behavioral homogenization is analogous to other forms of biotic homogenization such as taxonomic (increasing similarity in species composition), phylogenetic (declining spatial turnover in evolutionary lineages), functional (loss of functional trait diversity), and network (e.g., food webs/pollination networks converge) [7,8] homogenization. Hence, behavioral homogenization can be quantified as a decline in spatial and temporal ‘behavioral beta-diversity’ (i.e., the among-individual, -population, and -species variation in behavioral trait distributions across space and time). Signs of behavioral homogenization include the loss of geographic and temporal variation in behavior and the emergence of a common "syndrome" of behaviors among species that thrive in human-altered habitats (e.g., domestication syndrome) [9].

Indeed, urban environments around the globe seem to favor individuals and species with a particular suite of behavioral traits, so-called "urban wildlife syndromes,” characterized by increased boldness, reduced wariness, higher aggression, and greater willingness to exploit novel resources [10]. These patterns reflect the intense human presence in cities, which filters wildlife by favoring individuals and species that can tolerate frequent human disturbance and altered biotic and abiotic conditions. Moreover, urban populations often experience a neutral or positive balance in interactions with humans (e.g., animals may obtain food from humans and not be persecuted by humans), further reducing variance and promoting parallel shifts in behavior within and across species, which in turn amplifies community-level convergence.

## Evidence of urban-driven behavioral homogenization

**Fig 1:**
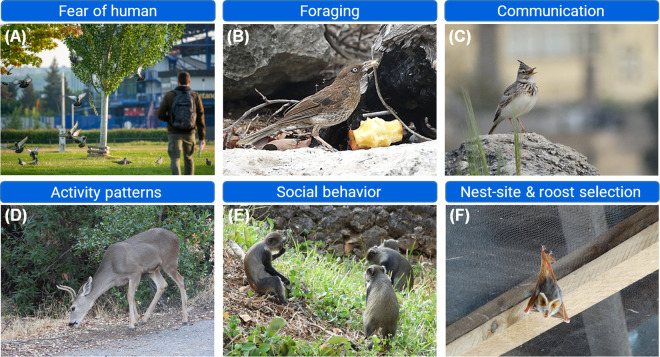
Examples of behavioral homogenization in urban areas. **A**. Urban animals generally exhibit reduced fear of humans. Within-species variance in this trait decreases among populations and over urbanization time [12–14]. **B**. Animals may increasingly rely on anthropogenic food subsidies such as refuse and feeders [15,16]. **C**. Birds in cities change characteristics of their acoustic communication to avoid overlap with low-frequency anthropogenic noise such as traffic [17]. **D**. Larger-bodied mammal species show the strongest shifts in their diurnal activity in urbanized habitats [18]. **E**. Species may exhibit an increasingly uniform behavioral profile in cities such as pronounced gregariousness and strong intraspecific territoriality [19,20]. **F**. Bats, birds, and other urban animals may increasingly use anthropogenic structures for breeding, roosting, perching, or resting [21,22]. Photo credit: Tomáš Jůnek (A), otherwise Peter Mikula.

Multiple lines of evidence indicate that urbanization drives behavioral homogenization across diverse taxa (Fig 1), either as replicated spatial convergence among independent cities or as temporal trajectories within urban populations characterized by increasing time since urbanization. These major lines of evidence include: antipredator behavior and fear of humans, foraging, communication, daily activity, social behavior, cognition and exploration, habitat use and movement, breeding-site choice and roosting, migration and seasonal patterns, and heterospecific interactions networks. To the best of our knowledge, these domains represent the best-studied behavioral axes along urban–rural gradients and thus provide the most comparable evidence. We note that behavioral homogenization may reflect phenotypic plasticity (habituation, learning, developmental effects) and/or heritable (mal)adaptive change. Because behavioral changes are often misattributed to adaptive evolution without evidence of heritable divergence and fitness benefits [11], we treat behavioral homogenization primarily as a phenotypic pattern unless such evidence is clearly demonstrated

### Antipredator behavior and fear of humans

One of the best documented and most consistent behavioral patterns observed is that animals in cities commonly exhibit reduced fear of humans (i.e., greater human tolerance) and other potential predators [13]. Urban conditions can erase behavioral differences among populations and species that are otherwise present across natural gradients. For example, European bird populations across many species show a clear latitudinal pattern in fear responses to humans in rural areas, with birds in Northern Europe being much more tolerant to human approach (i.e., had shorter escape distances) than those from Southern Europe [12]. However, that latitudinal trend virtually disappears across European cities. Moreover, within-species variation in fear responses was lower in urban populations when compared with geographically proximate rural populations [23]. In other words, the strong geographic variation in species fear of humans evident in natural settings is eliminated by urbanization, a pattern consistent with behavioral homogenization. Some evidence suggests that habituation-like processes, natural selection, and/or environmental filtering in cities can reduce behavioral plasticity and variation in animal fear responses in line with the time since species colonized urban environments [13,14]. For example, during the COVID-19 lockdowns (albeit rather short-term), urban birds in multiple countries did not substantially change their fear to humans and showed little further plastic adjustment [24].

### Foraging

Urban environments also impose convergent changes on feeding behavior across diverse species. Animals inhabiting cities often share a generalist and opportunistic foraging strategy [25]. Across continents, animals including passerines, gulls, and other birds [16], rats [26], raccoons [27], bears [15], and macaques [28], exhibit convergent foraging behaviors such as increased incidence of and reliance on feeding on anthropogenic food subsidies such as refuse and feeders, and even food begging. Even species that are naturally specialized can broaden their diets in cities and may converge on a more omnivorous, human-associated menu. For example, urban European coots (*Fulica atra*) readily reacted to bread-feeding by humans, a behavior not observed in rural coots, and this propensity was similar in multiple urban populations [29]. An increase in individual diet breadth can be interpreted as higher food diversity for that individual and species, yet at the among-individual and among-species levels it may reflect convergence on the same predictable, human-provided items (i.e., reduced dietary differentiation and increased overlap). Altogether, predictable anthropogenic food subsidies globally improve individual fitness and densities of opportunistic species, dampen temporal population variability, and simplify communities, fostering repeatable convergence on human-centered foraging strategies across species and regions [5].

### Communication

Organisms that communicate acoustically often adjust their signaling behavior in similar ways in noisy urban environments across many cities and species. For example, many urban songbird populations consistently sing at higher minimum frequencies than nearby non-urban conspecifics to avoid low-frequency anthropogenic noise such as traffic, a pattern first shown for great tits (*Parus major*) in Amsterdam and later replicated elsewhere, implying repeatable, directional change of the same trait across cities [30,31]. Urban birds also shift timing of their vocal activity to avoid having to communicate through the peak in anthropogenic noise, advancing or delaying song onset relative to rural populations, increasing their song amplitude (the Lombard effect), adjusting its temporal structure, narrowing its bandwidth, and exhibiting increased among-individual similarity in song syllable repertoires [32]. Noise also filters animal communities toward species with inherently higher-frequency signals, reinforcing cross-site parallelism via community composition. For example, acoustic masking by urban noise operates as a strong selective force that may disproportionately exclude larger species with lower frequency signals while smaller species may persist by transmitting higher-frequency signals [33]. Finally, some frequency shifts persist in common garden experiment conditions, indicating canalization beyond short-term plasticity and pointing to convergent evolution toward similar urban song phenotypes [34].

### Daily activity

Human activity patterns, such as diurnal schedules, artificial light at night (ALAN), and chronic noise, may synchronize, and therefore homogenize, wildlife daily routines. Across continents, mammals increasingly shift their activity toward nocturnality to avoid peak human presence [18]. ALAN advances dawn singing and foraging in birds, with stronger effects at higher illuminance, potentially synchronizing avian daily rhythms across cities [35]. In nocturnal taxa, ALAN may compress nocturnal activity by delaying the onset and advancing cessation [36,37]; ALAN expands twilight-like conditions and constricts true darkness [37]. These shifts may reduce among-population differences in activity timing and homogenize temporal niches, with communities converging on a small set of shared activity windows such as increased nocturnality/crepuscularity, where avoidance of human presence dominates. However, alternatively, ALAN may also disrupt sleep in free-living animals [38], and this may increase circadian oscillations and reduce repeatability in animal chronotypes [39,40], as well as prolonging and increasing overall animal activity [41]. Moreover, during the COVID-19 lockdowns, when human movement and transportation was severely limited in many regions, urban birds showed higher early-morning "activity" in citizen-science data, but this pattern was largely attributable to increased detectability in quieter cities, highlighting that apparent temporal convergence in behavior can partly reflect observational biases rather than true changes in the underlying activity patterns [42].

### Social behavior

A suite of social phenotypes may change in response to urban environments, including social structure (e.g., social interactions, aggression), social organization (e.g., group size, sex ratio), mating (e.g., sexual displays, mate choice, extra-pair behavior, mating systems), and parental care (e.g., coordination, offspring defense, food provisioning) systems [43]. For example, urban great tits have stronger territorial aggression than rural conspecifics [19], and urban striped field mice (*Apodemus agrarius*) were less likely to avoid close contact with conspecifics and exhibited greater tolerance during direct encounters with them compared with rural conspecifics [20]. There is evidence that cities may reduce among-individual variation in these behaviors. In Eurasian coots, urban populations, especially those that are longer established, exhibit uniformly bolder nest defense and higher aggression toward humans, whereas rural populations show higher variance, implying that urban filtering and/or selection canalizes coot responses over time [29]. In contrast, although territorial aggression in urban dark-eyed juncos (*Junco hyemalis*) dropped during the COVID-19 restrictions and remained below pre-anthropause levels for at least three years after reopenings, its variation in the study population actually increased during and after the lockdowns [44].

### Cognition and exploration

Cognition and exploration are central to urban colonization because cities repeatedly confront animals with novelty, favoring individuals and species with enhanced cognitive capabilities, behavioral flexibility, and problem-solving skills [45]. Populations of house mice (*Mus musculus*) subspecies with the longest commensal history with humans outperformed subspecies living commensally for shorter periods across multiple food-extraction tasks, and also showed lower among-individual variation in performance in set-ups requiring a single specific action, but not in set-ups that had multiple solutions [46]. In great tits, the level of neophobia (novel-object return latency) and its among-individual variation were lower in those in highly human-disturbed territories relative to birds in low-disturbance sites [47]. Similarly, urban great tits showed lower among-individual variation than their forest counterparts in exploratory behavior, but not in anti-predator and stress-related behaviors [48]. This highlights that urbanization can shift different behavioral axes in different (even opposing) directions, so broad generalizations about behavioral responses to cities should be treated cautiously.

### Habitat use and movement

Urbanization may force wildlife of disparate lineages into showing similar space-use patterns. Human activity patterns and altered landscapes in cities may concentrate diverse taxa into shared spatial niches and thereby flatten inter-individual differences in movement and home-range behavior. For example, in Illinois, USA, urban and suburban raccoons (*Procyon lotor*) maintained smaller home ranges than rural conspecifics, which, combined with sharply reduced seasonal shifts and spatial dispersion, suggests convergent spatial behavior and compressed individual variability that was driven by predictable and spatially clustered anthropogenic food resources [49]. Likewise, American white ibises (*Eudocimus albus*) that foraged exclusively at urban sites had the least-variable habitat use compared with individuals using natural sites or both habitat types; a finding consistent with constrained, convergent habitat use among urban specialists [50]. By contrast, tracking data collected during the COVID-19 lockdowns showed that reduced human mobility could rapidly alter mammal movement (such as how far and where individuals move to), but responses were highly heterogeneous across individuals, species, and contexts, highlighting that this was not a uniform shift in behavior [51].

### Breeding site choice and roosting

Cities can create structures with similar affordances as natural environments [52]. Bats, birds, and other urban dwellers may converge on the use of anthropogenic structures as breeding and roosting sites that are similar to natural tree cavities or cliffs. Many synanthropic bat species now breed, roost, or hibernate in buildings, bridges, culverts, or nest boxes [21]. Similarly, birds routinely occupy building ledges, attics, and under-bridge cavities in cities, and nesting in these novel locations may increase their reproductive success [22]. However, apparent homogenization in the use of ‘similar’ human-made structures can be misleading, because intrinsic differences in materials and geometry (e.g., nest-box insulation and design), structure-type specifics (e.g., bridge construction selecting for concrete girder designs), and heterogeneous urban lightscapes or ‘green’ substrates generate distinct microclimates and sensory environments despite superficial similarity [53,54]. Ultimately, we need more studies that compare within-species variance in breeding site choice along urban–rural gradients.

### Migration and seasonal patterns

Migrants are typically under-represented in cities, because urban environments filter communities toward resident species [25]. Moreover, cities reduce the costs of wintering because of the urban ‘heat-island effect’ and the increase in human-provided food (e.g., refuse and garden feeders). Therefore, migratory bird species that colonize urban areas frequently lose migratory behavior and become resident during urban colonization [55,56]. For example, urban European blackbirds (*Turdus merula*) repeatedly evolved greater sedentariness relative to forest conspecifics, with evidence for local adaptation and reduced migratory propensity in cities [57]. Supplementary feeding in urban areas facilitated the north-wintering expansion and potentially reduced migration tendency of Anna’s hummingbirds (*Calypte anna*) in North America [58]. Together, these results indicate that urban microclimate and predictable resources may impose consistent selection and filtering that canalize non-migratory tactics (e.g., via plasticity and rapid microevolution), driving cross-species and within-species convergence on residency and lowering among-population variance in movement strategies across landscapes. However, such homogenization effects may be trait-specific; for example, the breeding phenology of urban-breeding bird populations exhibits greater within-population variation in lay dates than non-urban conspecifics, likely reflecting fine-scale habitat heterogeneity within cities [59].

### Heterospecific interactions networks

Urban environments may also reshape interspecific interaction networks (so-called network rewiring). Because urban communities are typically species-poor and dominated by a recurring set of widespread, high-abundance keystone species [1], interaction networks in different cities may increasingly concentrate around the same few nodes, reducing network turnover and narrowing the diversity of interaction partners and behavioral pathways. For example, cities may promote heterospecific sociability (i.e., tolerance of, association with, or information use from other species) centered around a few species, with consequences for community organization and invasion dynamics. Heterospecific sociability can be consequential, since it may facilitate invasion of non-native species by providing social information, reducing predation risk, and improving foraging success [60]. Conversely, some invasive species can become interaction hubs that benefit natives, such as monk parakeets (*Myiopsitta monachus*), which act as nest-site facilitators because their communal nests are frequently occupied by ‘tenant’ species in both native and invaded ranges [61]. As we learn more about the value of these interaction networks, we may find that urban-driven changes have meaningful consequences for population viability.

## Implications of behavioral homogenization

**Fig 2:**
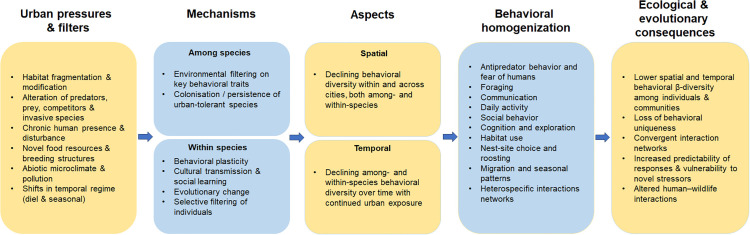
Schematic of urban-driven behavioral homogenization. Urban pressures and filters (left) act via various mechanisms (center and center left) to canalize among- and within-species variation in behavior both in space and time, ultimately homogenizing animal behavior among populations and species (center right), eroding behavioral diversity, and altering ecosystem functions and human–wildlife interactions (right).

The recognition of behavioral homogenization as a facet of global change biology carries several important implications (Fig 2). First, the loss of behavioral diversity across landscapes might reduce the ability of animal populations and communities to cope with further environmental changes [4]. If all urban wildlife shares a narrow range of behavioral traits, this can make animal communities more vulnerable to novel disturbances or less capable of performing certain ecological functions. For instance, widespread tameness might increase human–wildlife conflicts (e.g., vehicle collisions, physical attacks, property damage, zoonotic disease transmission, and spread of invasive species) or alter predator–prey interactions [6]. At the same time, the same suite of increasingly common traits, such as tolerance to humans or anthropogenic food use, are essentially the hallmarks of survivors in human-dominated landscapes. By identifying these traits (Fig 2), we can better predict which species might become winners or losers as urbanization and global human-driven environmental change progresses.

Second, there is also an evolutionary dimension: behavioral homogenization could be a precursor to genetic homogenization if the observed changes are heritable [62]. Over time, natural selection (or selective filtering of which individuals persist in cities) may lead to populations that are not only behaviorally similar but also genetically convergent in alleles underlying those behaviors. However, the same urban behavioral phenotype can arise via different mechanisms (genetic versus plastic change). Thus, distinguishing between the two mechanisms is crucial for conservation because they imply different vulnerabilities, limits to future responses, and management outcomes [11]. For example, plastic homogenization may be reversible if pressures change, whereas genetically based convergence can be more difficult to reverse and may entail trade-offs or maladaptation under future urban change.

Third, behavioral homogenization also implies the loss of unique behaviors and animal ‘cultures’, including locally specific cultural behavior that is fine-tuned to an environment, such as the tool use and vocal dialects seen in some birds and mammals [4,63–65]. As human influence spreads, we risk losing some of this behavioral diversity. In addition, certain culturally transmitted behaviors, such as vocal dialects, can have a critical role in species conservation and reintroduction success, especially for species where learned signals are crucial for mate choice, territory establishment, or group cohesion. In regent honeyeaters (*Anthochaera phrygia*), for example, population decline and isolation have led to the emergence of aberrant behaviors, which in turn significantly reduced pairing and nesting success; captive-bred individuals also developed atypical songs due to the absence of suitable adult tutors, jeopardizing their reintegration into wild populations [66].

Fourth, integrating behavior into biodiversity monitoring frameworks [67] could help us develop management and mitigation strategies. For example, by maintaining fear of predators in prey populations through managed predator presence, we could enhance survival in a homogenizing world; such interventions, however, can also trigger trophic cascades and shift movement, habitat use, foraging, and competition, so they require careful and context-dependent evaluation [68]. It is also important to preserve and restore natural behaviors where possible. For example, urban park designs that allow animals to express more natural foraging or anti-predator behavior can maintain some behavioral diversity in the city.

Fifth, urban-driven behavioral homogenization should not be assumed a priori. Its magnitude is likely trait-, taxon-, and region-dependent, because cities differ in urbanization history, anthropogenic disturbance regimes, resource provisioning, and habitat structure, and species and clades differ in constraints and plasticity. Comparative syntheses show that predictors of urban tolerance and trait filtering vary geographically. Moreover, some behavioral axes converge, whereas others remain variable or shift in different directions across urbanization gradients [10,25]. Documenting the diversity of outcomes is essential if we are to identify boundary conditions and moderators of homogenization. Moreover, this variation motivates the reporting of heterogeneity and null results alongside "positive" homogenization signals.

Finally, urban colonization can also be framed as behavioral diversification at the species level. For some taxa, city life represents an expansion of the realized niche or geographic range into a novel habitat type. Importantly, such apparent gains do not contradict behavioral homogenization in cities, because urban habitats are colonized non-randomly by individuals and species that share similar urban-tolerant traits and exploit recurring urban resources and structures. In other words, from a cross-city perspective, repeated spread of the same trait-filtered subset of individuals and species can therefore increase similarity among cities. Such similar urban pressures can drive both within- and among-species behavioral variance of urban dwellers toward a narrower set of responses across cities.

## Conclusions and future directions

Urban-driven homogenization in a variety of behavioral traits has been documented across multiple continents and unrelated species, indicating a multi-faceted process with true global-scale and cross-lineage consequences. Hence, urbanization can be viewed as a massive experiment replicating similar selective pressures (e.g., reduced predation, abundant human food, chronic anthropogenic disturbance, and artificial light and noise pollution) in cities around the world. The result is that wildlife in Los Angeles, Lima, Prague, Lagos, and Lahore tend to behave in similar ways despite vast differences in their natural histories and environments. Behavioral homogenization is thus emerging as a documented phenomenon, paralleling the well-known biotic homogenization of morphological, developmental, and other non-behavioral traits.

Researchers should explicitly incorporate behavior into homogenization studies. The key question is not only how much behavioral diversity exists, but whether the multivariate dispersion of behaviors across landscapes is shrinking under human pressure. Disentangling spatial and temporal as well as within- and among-species components requires experimental designs that combine replicated urban–rural contrasts across various taxa globally with gradients of time since urbanization and exposure to human disturbance or repeated longitudinal sampling of the same individuals, populations, and communities. In parallel, disentangling plasticity from heritable change requires moving beyond phenotype-only contrasts: robust inference about "urban adaptation" needs evidence of genetic divergence and fitness consequences, ideally via (multi-generation) common garden experiments, reciprocal transplants, or other approaches that jointly partition genetic versus environmental effects and quantify fitness [11]. Since available studies are strongly biased toward birds and mammals, future research should collate comparable behavioral metrics for other taxa, and embed them in standard diversity and turnover frameworks.

Technologies such as bio-logging, camera traps, automated acoustic recorders, and citizen science databases are making it increasingly feasible to gather behavioral data globally. Indeed, collaborative efforts have already compiled and analyzed massive datasets on avian fear responses to humans [12,69], song characteristics [70], and neophobia [71], providing raw material to analyze patterns of behavioral homogenization. Integrating these data will help determine the extent to which behavioral homogenization is occurring, and whether it is truly a global phenomenon or is more pronounced in regional or local contexts.

This perspective also complements established urban ecology frameworks on synanthropy (i.e., an ecological association with humans), synurbization (i.e., population-level adjustment to urban environments), and urban tolerance (i.e., persistence and habitat use along urbanization gradients) [25,72]. In this Essay, we placed these frameworks within a turnover-and-variance perspective, asking, beyond mean trait shifts, for example, whether cities repeatedly reduce and align behavioral variance across taxa and sites, and whether within-species variance in behavior is reduced or reshaped with continued urban exposure.

In conclusion, the homogenization of animal behavior is a novel and underappreciated aspect of biotic homogenization in the human-dominated era. Urbanization and other pervasive human pressures are not only making ecosystems more alike in terms of which species are present, but also in how those species behave. Recognizing behavioral homogenization as part of the broader biodiversity crisis expands the scope of both research and conservation agendas. It highlights the need to integrate behavior into global biodiversity monitoring frameworks, to design urban environments that accommodate a broader repertoire of behavioral expressions, and to consider cultural traits such as vocal dialects or tool use as conservation-relevant features. Addressing this blind spot in conservation research is essential for understanding organismal resilience, anticipating the ecological consequences of behavioral homogenization, and ultimately, for conserving the full complexity of life in the Anthropocene.

## Abbreviation:

ALAN: artificial light at night
